# Research on Small Square PCB Rogowski Coil Measuring Transient Current in the Power Electronics Devices

**DOI:** 10.3390/s19194176

**Published:** 2019-09-26

**Authors:** Chaoqun Jiao, Juan Zhang, Zhibin Zhao, Zuoming Zhang, Yuanliang Fan

**Affiliations:** 1 School of Electrical Engineering, Beijing Jiaotong University, No.3 ShangYuanCun, Haidian District, Beijing 100044, China; 17126087@bjtu.edu.cn (J.Z.); 18126182@bjtu.edu.cn (Z.Z.); 2 State Key Laboratory of Alternate Electrical Power System with Renewable Energy Sources, North China Electric Power University, No.2, Beinong Road, Changping District, Beijing 102206, China; zhibinzhao@ncepu.edu.cn; 3 Fujian Provincial Enterprise Key Laboratory of High Reliable Electric Power Distribution Technology, State Grid Fujian Electric Power Research Institute, No. 48 Fuyuan Branch, Cangshan District, Fuzhou 350007, China; fyl_fjdky@sina.cn

**Keywords:** PCB Rogowski Coil, Transient Current, power electronics devices, current measurement

## Abstract

With the development of China’s electric power, power electronics devices such as insulated-gate bipolar transistors (IGBTs) have been widely used in the field of high voltages and large currents. However, the currents in these power electronic devices are transient. For example, the uneven currents and internal chip currents overshoot, which may occur when turning on and off, and could have a great impact on the device. In order to study the reliability of these power electronics devices, this paper proposes a miniature printed circuit board (PCB) Rogowski coil that measures the current of these power electronics devices without changing their internal structures, which provides a reference for the subsequent reliability of their designs.

## 1. Introduction

With the development of semiconductor technology, a lot of power electronics devices such as IGBTs have been widely applied to high-power inverters, high voltage direct current high voltage direct current (HVDC) transmissions, and so on [1,2,3]. Therefore, in order to study the reliability of these devices, it is necessary to measure the distribution of the current in the device under different driving resistances, temperature conditions and layout modes [4,5,6]. However, commercially available current transformers and Rogowski coil current sensors are difficult to mount inside devices due to their large sizes. Moreover, such sensors are expensive, and large-scale use will undoubtedly increase cost. In [6] and [7], Bock B., Furuya M. et al. made a circular coil with a diameter of 19.7 mm and a rectangular coil with a side length of 24.7 mm using hand-wound coils, based on Rogowski coils. The self-integration working state measures the current of the internal chip of the press-pack IGBT device in different package forms. However, the bandwidth of the sensor is relatively narrow from the state of integration, and it is impossible to measure the current at a relatively low frequency. In [7], it is pointed out that a coil wound by hand is prone to uneven winding, which results in a difference in impedance between the coils and has a great influence on the measurement results.

With the development of PCB technology, the advantages of its digital wiring and fully automatic production have been able to overcome the interference factors caused by the winding method of a traditional coil. As a result, PCB technology has also been applied to the Rogowski coil. In [8,9], Gerber D. et al. proposed a new Rogowski coil with multiple PCB boards stacked to monitor the gate current outside the press-pack IGBT package. Tsukuda M. et al. proposed a new PCB Rogowski coil based on 6-layer PCB layout in the literature and applied it to the study of the current sharing characteristics of the welded IGBT module [10,11,12].

In this paper, a miniature, thin and low-cost Rogowski coil sensor is proposed for its boss geometry, without changing the internal structure of the device. The new current sensor can be placed inside the device, and the current variation during the opening process of the device under different driving modes is monitored. Simulation and experimental verification of the proposed sensor is carried out.

## 2. PCB Rogowski Coil Sensor

The Rogowski coil sensor is usually composed of a coil probe and an integrator. The coil and the integrator are connected by a coaxial cable.

### 2.1. The Working Principle of the Coil, the Establishment of the Equivalent Circuit Model and the Determination of the Working Mode

**Figure 1:**
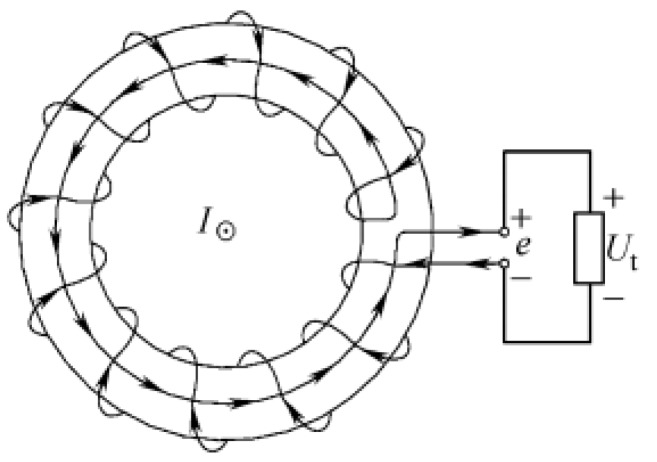
Basic Rogowski coil transducer.

As shown in Figure 1, the coil is uniformly wound on a non-magnetic skeleton of a constant cross-sectional area to form a closed loop, and the induced voltage generated in the coil is proportional to the rate of change of the current *I* passing through the center of the coil. The induced voltage is equivalent to [13]

(1) $$e=\mu_{0}NA\frac{dI}{dt}=M\frac{dI}{dt}$$

where *e* is the induced voltage (V) of the coil, *N* is the winding density (turns/m), *A* is the coil cross section (m2) and *M* is the mutual inductance (nH).

**Figure 2:**
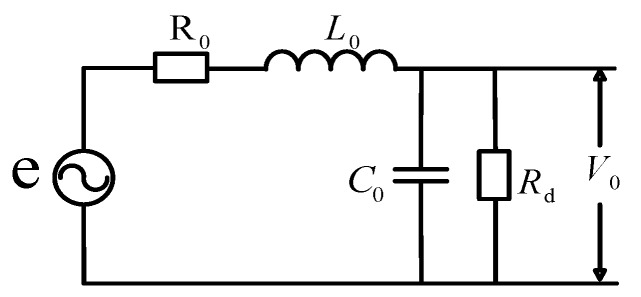
Equivalent circuit of coil termination.

The equivalent circuit of the coil is shown in Figure 2. The literature [14,15] points out that in a high-frequency state, the bandwidth of the coil is affected by the terminal impedance. When the current through the coil is in a centrally symmetrical position, the transfer function of the coil is [16,17,18]

(2) $$\frac{V_{0}}{e}=\frac{1}{1+\frac{j\omega L_{0}}{Z_{t}}-\omega^{2}L_{0}C_{0}}=\frac{1}{s^{2}L_{0}C_{0}+\frac{sL_{0}}{Z_{t}}+1}$$

where *V*_0_ is the terminal impedance voltage (V), *Z*_t_ is the terminal impedance (Ω), *L*_0_ is the coil inductance (H), *C*_0_ is the coil equivalent capacitance (F) and $C_{0}=4C/\pi^{2}$.

From the total parameter model of the upper atlas, when the terminal resistance value $R_{d}$ is small, there is $1/\omega C_{0}>>R_{d}$, $C_{0}$ close to the open circuit, so $i=i_{R}$; thereby

(3) $$e=M\frac{dI}{dt}=L_{0}\frac{dI}{dt}+(R_{0}+R_{d})I$$

When measuring a high-frequency current and satisfying $L_{0}\frac{dI}{dt}>>(R_{0}+R_{d})I$, Equation (3) can be changed into

(4) $$e=M\frac{dI}{dt}\approx L_{0}\frac{dI}{dt}$$

Further

(5) $$V_{0}=IR_{d}\approx \frac{M}{L_{0}}R_{d}I$$

It can be seen from the above formula that the coefficient is constant, and the output voltage of the coil is proportional to the current being measured. In this case, no external integration circuit is required, so the process is called self-integration.

Conversely, when the frequency change is not high and the terminating resistance $R_{d}$ is large, Equation (3) can be reduced to

(6) $$e=M\frac{dI}{dt}=(R_{0}+R_{d})I$$

and then

(7) $$V_{0}=IR_{d}=M\frac{R_{d}}{\left(R_{0}+R_{d}\right)}\frac{dI}{dt}$$

(8) $$I=\frac{\left(R_{0}+R_{d}\right)}{MR_{d}}\int V_{0}dt$$

It can be seen that it is necessary to integrate the output voltage to obtain the measured current, which is the external integral working mode of the Rogowski coil.

The inductance and capacitance of the coil are obtained by the formula

(9) $$L_{0}=\mu_{0}N^{2}lA=MN_{t}$$

(10) $$C_{0}=\frac{4\pi \varepsilon_{0}\varepsilon_{r}l}{\ln(A/a)}$$

where *l* is the length of the coil (m), *a* is the cross-sectional area (m2) of the loop inside the skeleton $\varepsilon_{r}$, $\varepsilon_{r}\;$ is the relative dielectric constant of the skeleton material and Nt is the total number of turns of the coil (turn).

The natural angular frequency of the coil $\omega_{0}$ is

(11) $$\omega_{0}=\frac{1}{\sqrt{L_{0}C^{\prime }}}=2\pi \frac{1}{\sqrt{L_{0}C_{0}}}$$

The transfer function of the coil output voltage and the current I through the coil can be obtained by simplifying Equations (1) and (2) [18]:

(12) $$\frac{V_{0}}{I}=\frac{sM}{s^{2}L_{0}C_{0}+\frac{sL_{0}}{Z_{t}}+1}=\frac{sM}{1+2\xi T_{c}s+T_{c}{}^{2}s^{2}}$$

The equivalent coil delay is $T_{C}=\sqrt{L_{0}C_{0}}$, and the damping coefficient is $\xi =\left(\sqrt{L_{0}/C_{0}}\right)/\left(2Z_{t}\right)$.

In order to increase the sensitivity and bandwidth of the coil sensor, it is necessary to increase the mutual inductance M and the pass band of the coil. The improvement of the mutual inductance is mainly reflected in the increase of winding density and cross-sectional area, and the increase of M will lead to a decrease of the resonant frequency of the coil. Since the PCB coil needs to be nested inside the IGBT module, the improvement space is limited; The improvement is mainly in the two aspects of the coil’s own resonant frequency and the optimization of the integrator. However, the widening of the bandwidth is mainly for the improvement of the integrator, and when the coil works outside the integral, the terminal resistance is not limited. In order to measure the IGBT current well, the external integral working mode is chosen.

### 2.2. Error Analysis of Rogowski Coil

**Figure 3:**
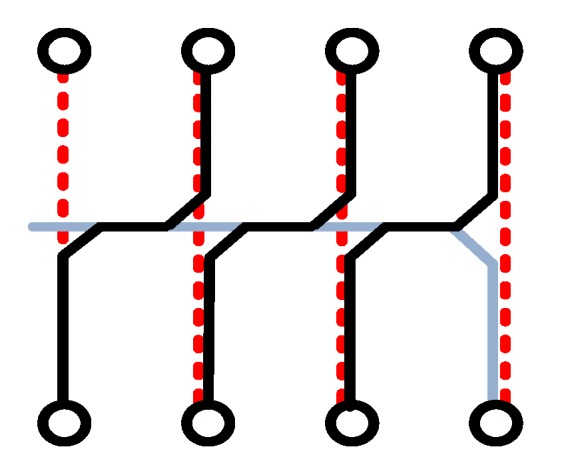
Fishbone with return line.

It is worth mentioning that this paper adopts a new type of winding method that can effectively reduce the interference of external signals on the coil, as shown in Figure 3.

The PCB coil is wound around a circle. Since the small coil is in a straight line, it can be regarded as forming a large line. This article wraps around a large line and back line at the end of the winding.

When the disturbing magnetic field is parallel to the conductor to be tested, the law of electromagnetic induction dictates that the two coils will generate an equal and opposite induced electromotive force since the large coil is connected in series with the return line in the opposite direction. Thus, the lines will cancel each other out.

When the interfering magnetic field is perpendicular to the conductor to be tested, the interfering magnetic field generates an induced electromotive force of equal magnitude but opposite direction on the small line where the Rogowski coil is symmetric, thus canceling each other and eliminating the interference of the magnetic field in the current measurement.

In addition, the return line can also effectively reduce the error caused by the eccentricity of the coil and the conductor under test, which will not be elaborated upon in this paper.

### 2.3. Parameters and Frequency Characteristics of Rogowski Coil

Combined with the above analysis, the final selected square coil parameters are as Table 1.

**Figure 4:**
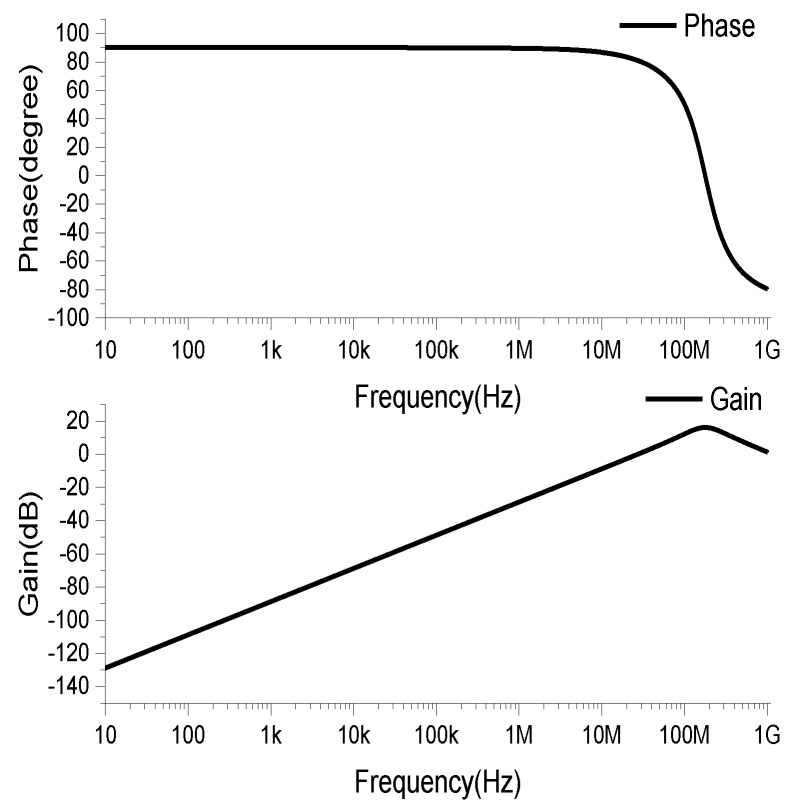
Frequency characteristic of coil.

The coils were drawn by AD (Altium Designer) software, and the drawn PCB coils were imported into the Ansys Q3D Extractor for parameter extraction. The equivalent circuit model was built in the PSpice simulation software, and the frequency characteristics of the coil are shown in Figure 4. In the frequency band of 10Hz–1MHz, the amplitude gain increases linearly with the increase of the frequency, and the phase offset is 90°. This is a typical differential operation mode, which requires the subsequent signal circuit to integrate the differential of the measured current. In order to obtain a constant amplitude gain in this band, it is also necessary to properly configure the parameters of the subsequent integration circuit.

### 2.4. Subsequent Integration Circuit Design

**Figure 5:**
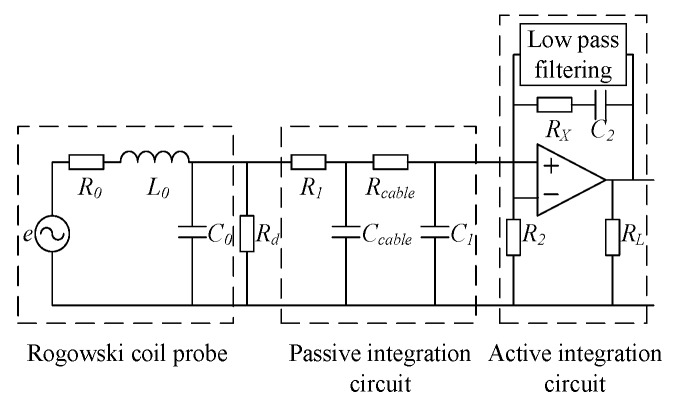
Lumped parameter model of the Rogowski transducer.

The integrator of the Rogowski coil sensor contains a passive integral link and an active integral link, as shown in Figure 5. The coil works in differential mode, *R*_1_ and *C*_1_ form a passive integral link and *R*_2_, *C*_2_ and an operational amplifier form a low-frequency active integral link [17,18,19,20,21]. In the mid-band, the integral action is achieved by the passive *R*_1_*C*_1_ network, since $1/\left(\omega C_{2}\right)\ll R_{2}$ and the op amp appears as a unity gain amplifier. In the low band, the passive *R*_1_*C*_1_ network exhibits unity gain, at which time constant *R*_2_*C*_2_ decided by the active inegration. The link realizes the integral function.

In the design process of the integrator circuit, the parameters are designed according to the sensitivity *R_sh_* required by the Rogowski coil sensor.

In order to ensure that the Rogowski coil sensor has a constant sensitivity *R_sh_* within the specified operating frequency range, the time constants of the passive and active integration need to be matched. The transfer function of the entire passive and active integration network is

(13) $$\frac{V_{out}}{V_{t}}=\frac{\left(1+T_{i}s\right)}{T_{i}s\left(1+T_{0}s\right)}$$

Among them, $T_{i}=C_{2}R_{2}$ and $T_{0}=(C_{1}+C_{cable})R_{1}$. C_cable_ is the equivalent capacitance of the coaxial cable.

By matching the the time constant of the passive integration with the time constant of the active integration $T_{i}=T_{0}$,

(14) $$V_{out}=\frac{1}{T_{i}}\int V_{t}dt$$

Combining Equations (3) and (13)–(15), the transfer function of the Rogowski coil sensor is obtained.

(15) $$\frac{V_{out}}{I}=\frac{R_{sh}\cdot e^{-j\theta_{c}}}{\left(1+2\xi T_{c}s+T_{c}{}^{2}s^{2}\right)\left(1+T_{b}s\right)}$$

where $R_{sh}=M/T_{i}$ and $T_{b}=1/\left(2\pi \times GBW\right)$.

**Figure 6:**
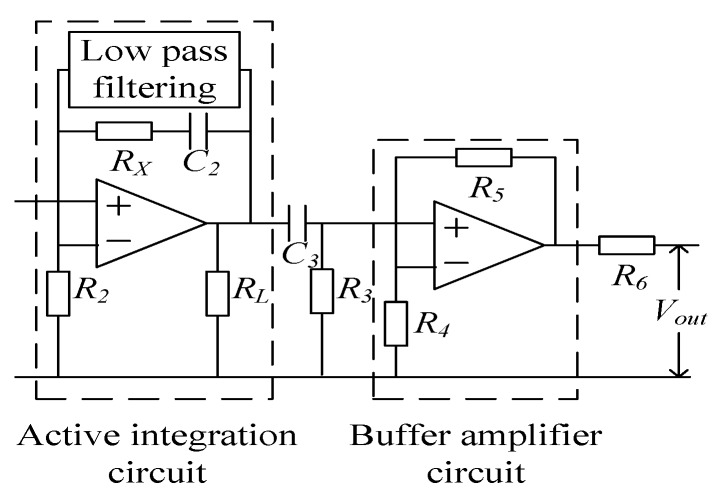
Optimization of integral circuit.

In order to filter out the low-frequency noise introduced by the operational amplifier in the active integration section, a high-pass filter circuit can be added after the active integration step. In order to reduce the gain of the active integral link and improve the response of the sensor to the rate of change of the measured current di/dt, an amplifying circuit can be added to change the gain of the amplifying circuit by adjusting the corresponding resistance value, as shown in Figure 6.

**Figure 7:**
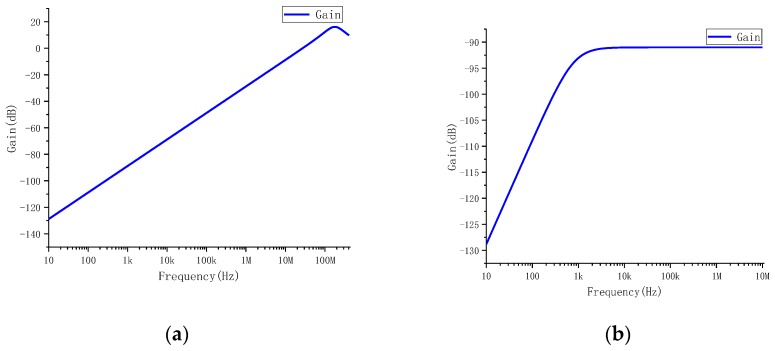

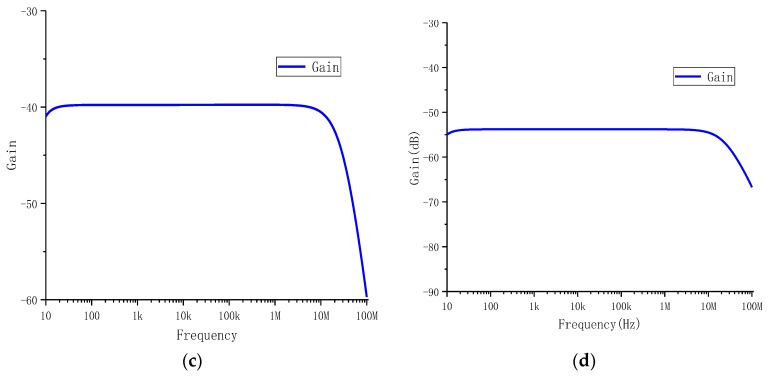
Amplitude–frequency characteristics of the PCB coil sensor: (**a**) amplitude–frequency characteristics, (**b**) amplitude–frequency characteristics with passive integral, (**c**) amplitude–frequency characteristics with active integral and (**d**) overall amplitude–frequency characteristics.

The amplitude–frequency characteristics of the PCB coil sensor are shown in Figure 7a–d.

## 3. Simulation and Experimental Verification

### 3.1. Simulation of PCB Rogowski Coil

**Figure 8:**
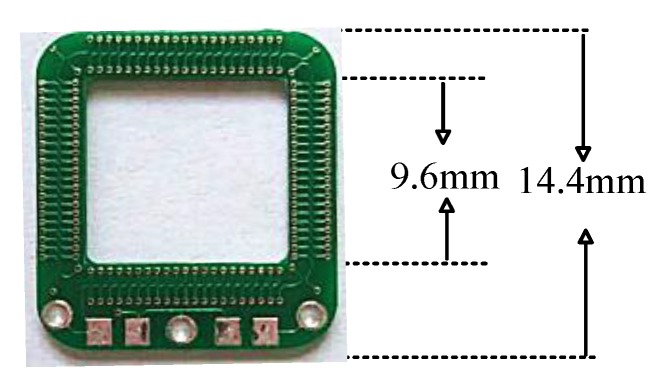
A PCB Rogowski coil.

The PCB Rogowski coil made in this paper is depicted by the physical map and coil size shown in Figure 8.

**Figure 9:**
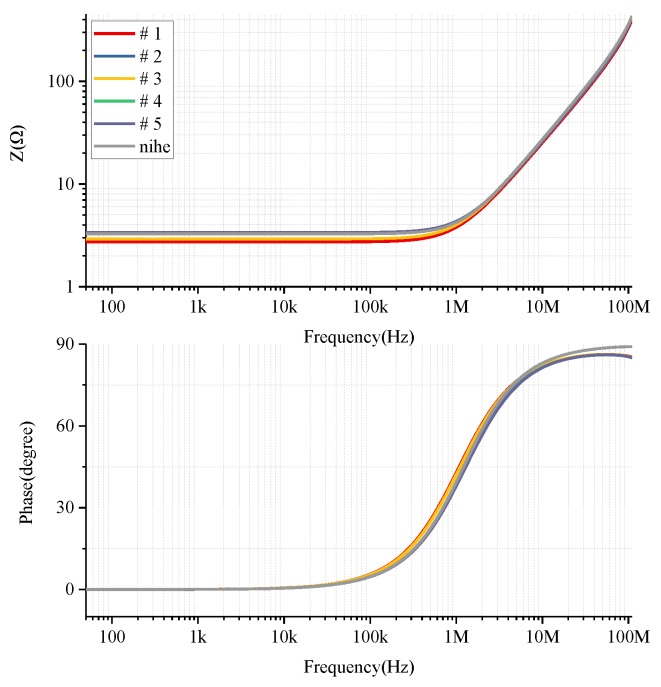
Comparison of impedance characteristics of the coil measured by simulation and experiment.

Using the impedance analyzer Agilent 4294 A, the impedance parameters of the PCB Rogowski coils shown in Figure 8 were tested to obtain the impedance frequency characteristics of each coil probe (Figure 9). Compared with the results of PSpice simulation processing, it can be seen that the simulation results were basically consistent with the experimental test results in a wide frequency range, indicating the accuracy of the equivalent circuit model in this frequency range.

As can be seen from Figure 2, the coil probe impedance–frequency characteristic *Z*(*s*) is

(16) $$Z\left(s\right)=(r+Ls)//\frac{1}{Cs}=\frac{r+Ls}{1+rCs+LCs}$$

**Figure 10:**
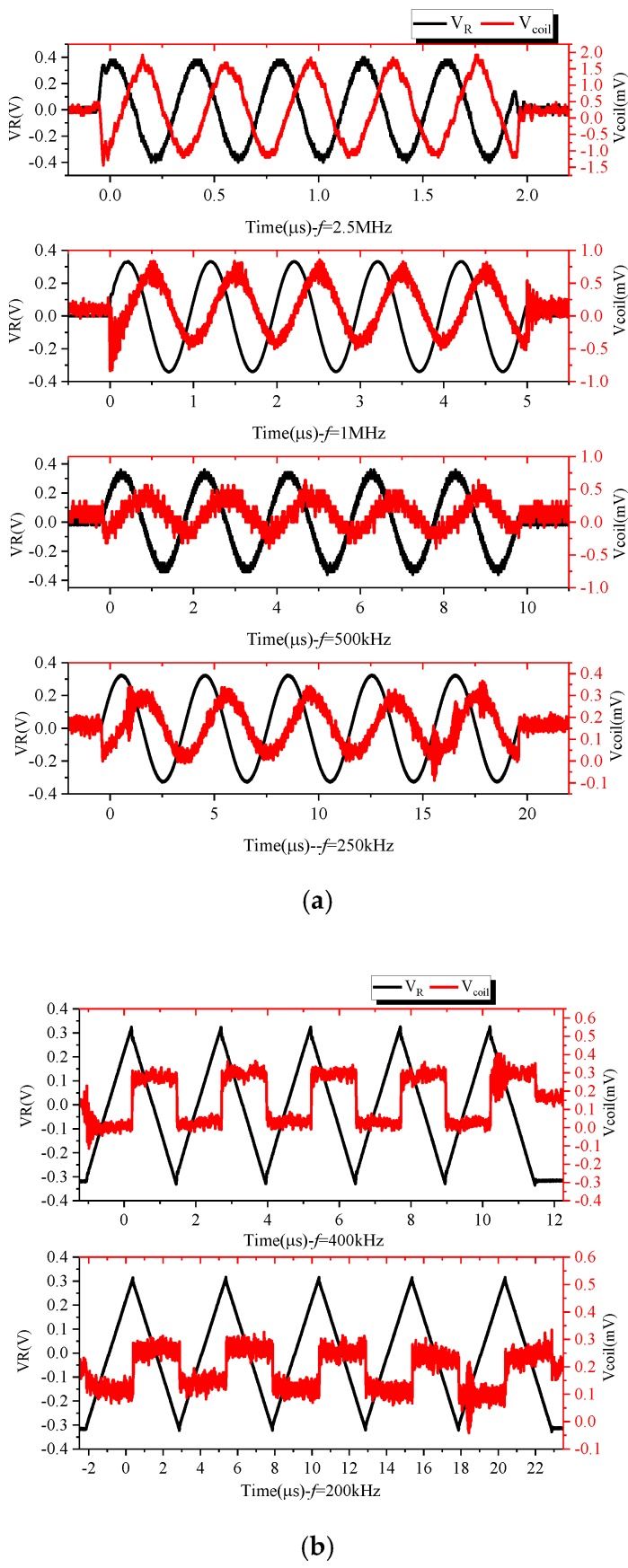
Roche coil differential relationship verification: (**a**) 2.5 MHz, 1 MHz, 500 kHz, 250 kHz sinusoidal and Rogowski coil outputs; (**b**) 400 and 200 kHz triangular wave and Rogowski coil outputs.

The impedance parameters of each coil are shown in Table 2. Due to the bandwidth limitations of the impedance analyzer, impedance characteristics only at 40–110 MHz could be measured. It can be seen in Figure 10a that the resonant frequency of the coil probe was greater than 110 MHz, which provides a possibility for subsequent expansion of the bandwidth. The frequency characteristics of the coil are given above. In combination with the existing experimental conditions, signals of different frequencies were generated by the signal generator and output via the power amplifier. The PCB Rogowski coil was serially connected to the current-carrying conductor, and the voltage signal across the terminal resistance was measured and compared with the measured signal. The output of the 2.5 MHz, 1 MHz, 500 kHz and 250 kHz sinusoidal signals and 400 and 200 kHz triangular wave signals is shown in Figure 10. In Figure 10a, the output of the Rogowski coil is cosine, and there is a phase difference of 90° from the input; the output of the Rogowski coil in Figure 10b is a rectangular wave. Both of these verify the differential operation of the Rogowski coil.

**Figure 11:**
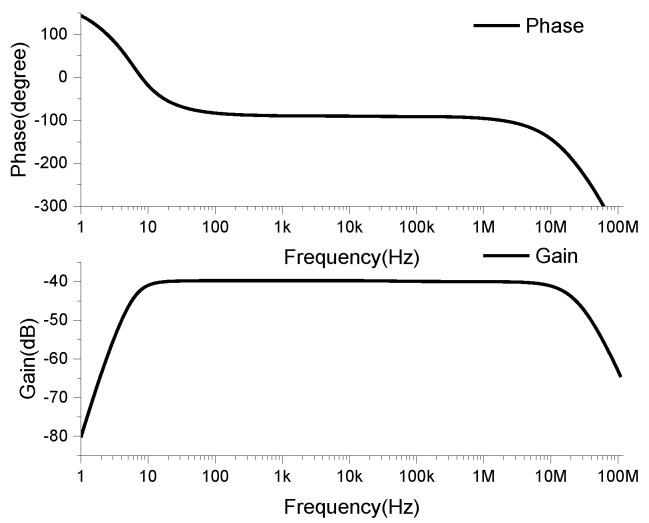
Rogowski transducer Bode diagram.

The coil and integrator were connected by a coaxial cable of 100 pF/m, and the frequency characteristics of the Rogowski coil sensor were obtained by simulation, as shown in Figure 11.

### 3.2. Experimental Verification of the PCB Rogowski Coil Current Transformer

The switching characteristics of many power electronics devices have a large current change rate in a short switching time. The circuit for testing the characteristics of the diode-clamped inductive load circuit is not completed yet, since the lightning current wave also has the characteristic of a sudden change in a short time. As a result, this section takes the detection of an 8/20-μs standard lightning current wave as an example to verify the performance of the self-designed PCB Rogowski coil current transformer. The main parameters of the device are shown in Table 3.

**Figure 12:**
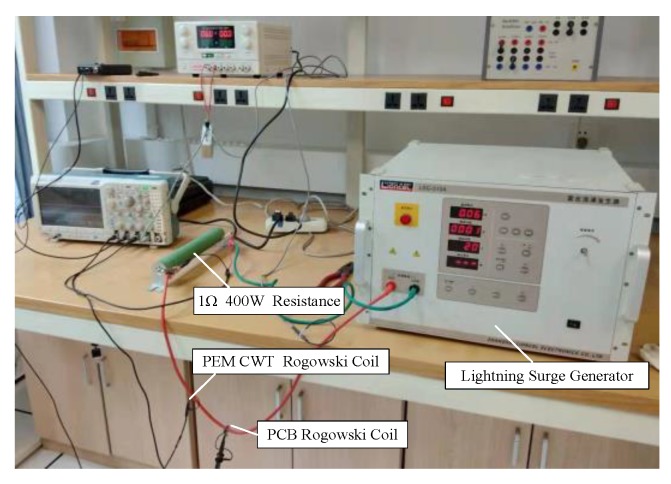
Scene of the lightning surge test.

The output of the lightning surge generator was connected to a 1Ω 400W resistor. The PCB Rogowski coil and the PEM CWT Rogowski coil (see Table 4 for parameters) were passed through the wire containing the output signal of the lightning surge generator to continuously change the lightning surge. The output voltage amplitude of the device recorded and saved the waveform data of the oscilloscope. The experimental site is shown in Figure 12.

**Figure 13:**
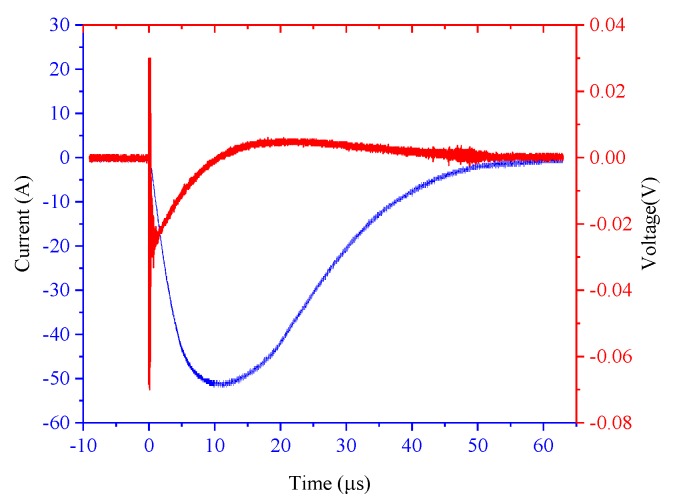
Mutation characteristics of the PCB Rogowski coil.

In this experiment, the output voltage amplitude of the lightning surge generator ranged from 300 to 550 V, and was recorded every 50 V to obtain six sets of experimental waveforms. The channel connected to the PEM CWT Rogowski coil during the experiment set the attenuation ratio so that the instantaneous value of the current did not exceed the maximum range of the oscilloscope current probe. As shown in Figure 13, taking the peak current of the measured current as 400 A, red represents the output waveform of the PCB Rogowski coil probe, and blue represents the measurement result of the PEM CWT Rogowski coil. The output waveform of the PCB Rogowski coil probe produces a significant abrupt change at the moment of sudden change of the measured current signal. After rising to the maximum value, it gradually decreases. After zero-crossing, it increases inversely and then gradually decays to 0. Here again, the PCB Rogowski coil is verified by the differential way of working.

**Figure 14:**
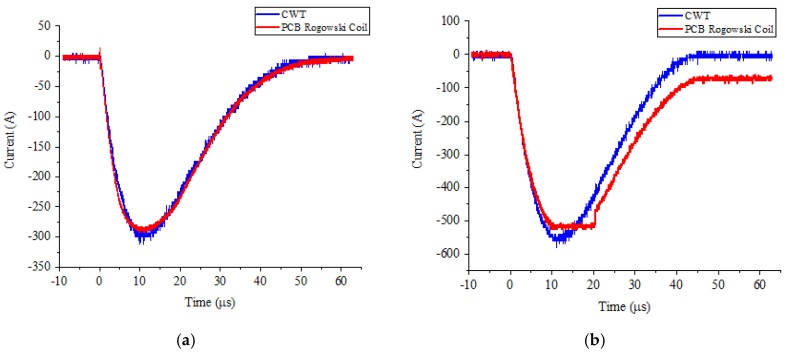
Experimental results of lightning surge signal (negative polarity). (**a**) The measured current peak value is 300 A. (**b**) The measured current peak value is 550 A.

The measurement results are shown in Figure 14 for the output voltage amplitude of the lightning surge generator at 300 and 550 V. When the measured current peak value was 300 A, the output waveform of the PCB Rogowski coil sensor and PEM CWT Rogowski coil were basically the same; since the sensitivity of the sensor was 10 mV/A, the internal circuit configuration of the operational amplifier determined that the amplitude of the output signal did not exceed the supply voltage. In this experiment, the power supply voltage of the op amp was ±5 V DC, meaning that the measured current amplitude of the PCB Rogowski coil sensor was notmore than 500 A. Therefore, when the peak value of the measured current increased to 550 A, the output waveform of the PCB Rogowski coil sensor would be distorted. If you want to measure a higher current amplitude, you can consider both the sensitivity of the sensor or the supply voltage of the op amp.

## 4. Conclusions

In this paper, a small PCB Rogowski coil sensor was designed for power electronics devices. The design flow was introduced in detail. The differential action of the Rogowski coil was verified using both a simulation and an experiment. The feasibility of the designed Rogowski coil was verified, which provides a reference for the reliability research and optimization design of subsequent devices.

## Figures and Tables

**Table 1:** PCB coil parameter.

Turns	Inside	Outside
84	9.6 mm	14.4 mm
Minimum aperture	Plate thickness	Trace width
0.2 mm	1 mm	3.5 mil

**Table 2:** Impedance parameters of the PCB Rogowski coil.

Serial Number	#1	#2	#3	#4	#5
Internal resistance *r* (Ω)	4.28	4.14	4.07	4.85	4.63
Self-induct *L* (nH)	439	445	436	440	441
Mutual sense *M* (nH)	6.10	6.18	6.06	6.11	6.13

**Table 3:** Main parameters of lightning surge generator (LSG-510A).

Output Waveform	Integrated Waveform	
	Open Circuit Voltage Wave	Short Circuit Current Wave
cutting edge	1.2 μs ± 30%	8 μs ± 20%
Pulse width	50 μs ± 20%	20 μs ± 20%
Peak	0~6 kV ± 10%	0~3 kA ± 10%
Output impedance	2Ω ± 10%	

**Table 4:** Main parameters of the CWT Rogowski coil transducer.

Current Peak (A)	d*i*/d*t* (kA/μs)	Bandwidth
600	40	6.2–30 M
Sensitivity	Attenuation characteristic	Maximum noise
10	6	10

## References

[1] Shigekane H., Kirihata H., Uchida Y. Developments in modern high power semiconductor devices. Proceedings of the 5th International Symposium on Power Semiconductor Devices and ICs.

[2] Chokhawala R., Danielsson B., Angquist L. Power semiconductors in transmission and distribution applications. Proceedings of the 13th International Symposium on Power Semiconductor Devices & ICs. IPSD ’01 (IEEE Cat. No.01CH37216).

[3] Volke A., Wendt J., Hornkamp M. (2012). IGBT Modules: Technologies, Driver and Application.

[4] Müsing A., Ortiz G., Kolar J.W. Optimization of the current distribution in press-pack high power IGBT modules. Proceedings of the 2010 International Power Electronics Conference—ECCE ASIA-.

[5] Tang X., Cui X., Zhao Z., Zhang P., Wen J., Zhang R. (2017). Analysis of Transient Current Distribution Characteristics of Parallel Chips in Press Pack IGBT. CSEE.

[6] Bock B., Krafft E.U., Steimel A. (2003). Measurement of multiple chip currents in a Press-Pack IGBT using Rogowski coils. Eur. Power Electron. Conf..

[7] Furuya M., Ishiyama Y. (2002). Current measurement inside press pack IGBTs. Fuji Electr. J..

[8] Gerber D., Guillod T., Biela J. IGBT gate-drive with PCB Rogowski coil for improved short circuit detection and current turn-off capability. Proceedings of the 2011 IEEE Pulsed Power Conference.

[9] Gerber D., Guillod T., Leutwyler R., Biela J. (2013). Gate Unit with Improved Short-Circuit Detection and Turn-Off Capability for 4.5-kV Press-Pack IGBTs Operated at 4-kA Pulse Current. IEEE Trans. Plasma Sci..

[10] Tsukuda M., Koga M., Nakashima K., Omura I. (2016). Micro PCB Rogowski coil for current monitoring and protection of high voltage power modules. Microelectron. Reliab..

[11] Koga M., Tsukuda M., Nakashima K., Omura I. Application-specific micro Rogowski coil for power modules-Design tool, novel coil pattern and demonstration. Proceedings of the CIPS 2016 9th International Conference on Integrated Power Electronics Systems.

[12] IXYS Is Now Part of Littelfuse. http://www.westcode.com/.

[13] Rogowski W., Steinhaus W. (1912). Die Messung der magnetischen Spannung. Arch. Für Elektrotechnik.

[14] Cooper J. (1963). On the high-frequency response of a Rogowski coil. J. Nucl. Energy Part C Plasma Phys. Accel. Thermonucl. Res..

[15] Ray W.F., Hewson C.R., Metcalfe J.M. High frequency effects in current measurement using Rogowski coils. Proceedings of the 2005 European Conference on Power Electronics and Applications.

[16] Ray W.F., Davis R.M. (1993). Wide Bandwidth Rogowski Current Transducers: Part I: The Rogowski Coil. EPE J..

[17] Ray W.F. (1993). Wide Bandwidth Rogowski Current Transducers: Part II: The Integrator. EPE J..

[18] Ray W.F., Hewson C.R. High performance Rogowski current transducers. Proceedings of the Conference Record of the 2000 IEEE Industry Applications Conference.

[19] Hewson C.R., Ray W.F., Metcalfe J. Optimising high frequency integrator operation of rogowski current transducers. Proceedings of the 2007 European Conference on Power Electronics and Applications.

[20] Ray W.F. (2003). Current Measuring Device. U.S. Patent.

[21] Wang B., Wang D., Wu W. (2009). Frequency Response Analysis of a Rogowski Coil Transducer and Its Design Method. Trans. China Electrotech. Soc..

